# Serum zinc concentration and quality of life in chronic liver diseases

**DOI:** 10.1097/MD.0000000000018632

**Published:** 2020-01-03

**Authors:** Hiroki Nishikawa, Hirayuki Enomoto, Kazunori Yoh, Yoshinori Iwata, Yoshiyuki Sakai, Kyohei Kishino, Naoto Ikeda, Tomoyuki Takashima, Nobuhiro Aizawa, Ryo Takata, Kunihiro Hasegawa, Noriko Ishii, Yukihisa Yuri, Takashi Nishimura, Hiroko Iijima, Shuhei Nishiguchi

**Affiliations:** Division of Hepatobiliary and Pancreatic disease, Department of Internal Medicine, Hyogo College of Medicine, Nishinomiya, Hyogo, Japan.

**Keywords:** BDI-II, chronic liver disease, PSQI-J, SF-36, zinc

## Abstract

Health related quality of life (HRQOL) in chronic liver disease (CLD) patients has been attracting much attention these days because it is closely associated with clinical outcomes in CLD patients. HRQOL has become established as an important concept and target for research and practice in the fields of medicine. A critique of HRQOL research is the lack of conceptual clarity and a common definition of HRQOL. Using a clear definition of HRQOL may increase the conceptual understanding. In this study, we aimed to elucidate the association between serum zinc (Zn) level and HRQOL as assessed by the Beck Depression Inventory-2nd edition (BDI-II), Pittsburgh Sleep Quality Index Japanese version (PSQI-J) and the 36-Item Short Form Health Survey (SF-36) in CLD patients (n = 322, median age = 65 years, 121 liver cirrhosis (LC) patients (37.6%)). The median serum Zn level for all cases was 73.2 μg/dl. The median BDI-II score and PSQI-J score were 6 and 5, respectively. Patients with higher BDI-II score tended to have lower serum Zn level compared with those with lower BDI-II score. Similar tendencies were observed in patients with higher PSQI-J score. In the SF-36, physical functioning, role physical and physical component summary score significantly correlated with serum Zn level regardless of age, liver disease etiology and the LC status. While mental health and mental component summary score did not significantly correlate with serum Zn level regardless of age, liver disease etiology and the LC status. In conclusion, serum Zn level can be a useful marker for decreased HRQOL in patients with CLDs, especially for physical components.

## 1 Introduction

Zinc (Zn) is widely distributed in the human body and it is an important trace element that is required for normal cell development, proliferation and differentiation.^[1]^ It is also well known to be crucial to ensure an appropriate immune response such as anti-inflammatory effects or antioxidant effects and to be a critical cofactor for ammonia metabolism.^[2–5]^ Serum Zn loss can result in a wide spectrum of clinical manifestations including impaired taste and smell, appetite loss, anemia, body hair loss, atrophy of testis, pressure ulcer, cerebral and immune dysfunction, and impairment of drug excretion ability and they are frequently recognized in chronic liver diseases (CLDs) because Zn homeostasis is mainly regulated in the liver.^[4,6–10]^ The degree of Zn loss has been shown to correlate well with the severity of liver diseases.^[11,12]^ The possible causes of low serum Zn levels in advanced liver cirrhosis (LC) patients are considered to be poor dietary intake, excessive urinary losses, and insufficient intestinal absorption.^[13]^ A previous randomized controlled trial (RCT) reported that Zn replenishment therapy can be safe and effective for treating hyperammonemia in LC patients.^[11]^ However, a lot of clinical aspects of Zn loss have not yet been clarified in CLD patients.

Health related quality of life (HRQOL) in CLD patients has been attracting much attention these days because it is closely associated with clinical outcomes in CLD patients.^[14–17]^ Increasing number of pivotal clinical trials have adopted not only survival as primary endpoint but also HRQOL as additional study endpoints.^[14,15]^ Depressive state or sleep disorder can affect HRQOL in CLD patients. Depression is an essential neurocognitive symptom in CLD patients.^[18]^ The Beck Depression Inventory-2nd edition (BDI-II) is one of representative screening tools for depression.^[19]^ While CLD patients frequently describe sleep problems. Sleep disorders negatively impact innate immunity and are commonly associated with neurocognitive alterations in CLD patients.^[20]^ Currently, one of well validated patient-reported sleep questionnaires with extensive use is Pittsburgh Sleep Quality Index (PSQI).^[21]^ On the other hand, HRQOL in CLD patients can be assessed by the 36-Item Short Form Health Survey (SF-36).^[22]^ However, to our knowledge, the association between serum Zn level and HRQOL as assessed by BDI-II, PSQI and SF-36 in CLD patients is not well understood. In this study, we sought to elucidate these issues.

## 2 Patients and methods

### 2.1 Patients

A total of 322 CLD patients with data for BDI-II, PSQI (Japanese version, PSQI-J), SF-36 and serum Zn level were admitted to Hyogo college of medicine hospital between December 2013 and August 2018 and were analyzed in the current study. LC diagnosis was based on histological findings, laboratory data and/or imaging findings in each analyzed subject.

### 2.2 BDI-II, PSQI-J score and SF-36

The BDI-II is a globally accepted screening tool for assessing the severity of depression.^[23,24]^ The BDI-II has good psychometric properties, internal consistency and high reliability. The BDI-II is a self-administered questionnaire that comprises 21 items, and each answer is evaluated on a four-point scale (0 to 3 points).^[23,25]^ Higher BDI-II score suggests a more serious depression state. Our study subjects were categorized as normal (BDI-II score: 0–10 point), and the severity of depression state as minimal (BDI-II score: 11–16 point), mild (BDI-II score: 17–20 point), moderate (BDI-II score: 21–30 point) and severe (BDI-II score ≥31 point).^[24,26–28]^ Because mild, moderate and severe depression state are considered to be clinically meaningful depression state, our study subjects were divided into two groups: patients with normal or minimal depression (group A: BDI-II score, 0–16 point) and patients with mild, moderate or severe depression (group B: BDI-II score, ≥17 point).

Sleep quality was evaluated by PSQI-J as a screening tool for sleep disorder.^[21,29,30]^ This questionnaire consists of 10 queries that form 7 categories: sleep duration, subjective sleep quality, sleep latency, sleep disorders, habitual sleep efficiency, usage of sleep medications and daytime disturbance. Each category is rated on a scale of 0 to 3 (the sum of PSQI-J scores for all categories is 21 point at the maximum). Higher PSQI-J score indicates a poorer sleep quality. Patients with PSQI-J score 0–5 point were defined as normal, those with PSQI-J score 6–8 point as mild sleep disorder, those with PSQI-J score 9–11 point as moderate sleep disorder and those with PSQI-J score ≥12 point as severe sleep disorder.^[21,29,30]^ Our study subjects were divided into two groups: patients with normal or mild sleep disorder (group C: PSQI-J score, 0–8 point) and patients with moderate or severe sleep disorder (group D: PSQI-J score, ≥9 point).

Study subjects were also asked to complete the Japanese version of the SF-36 (self-reported questionnaire). It consists of 36 items and is classified into multi-item (8 items) scales: physical functioning (PF), role physical (RP), bodily pain (BP), general health perception (GH), vitality (VT), social functioning (SF), role emotion (RE) and mental health (MH).^[31]^ The physical component summary score (PCS) and the mental component summary score (MCS) are also included in this questionnaire.^[31]^ Thus, a total of 10 items were included in a questionnaire.

The association between serum Zn level and BDI-II score, PSQI-J score and SF-36 were examined. The ethics committee meeting of our hospital acknowledged this study (approval no. 2296). The protocol in the study rigorously observed all regulations of the Declaration of Helsinki.

### 2.3 Statistical considerations

In continuous parameters, Student's *t* test, Mann-Whitney *U* test or Pearson's correlation coefficient *r were employed to assess group difference*, as applicable. Unless otherwise mentioned, data were presented as median value (interquartile range (IQR)). The threshold for statistical significance was considered as *P* < .05. We employed the JMP 14 (SAS Institute Inc., Cary, NC) to analyze statistically.

## 3 Results

### 3.1 Patient baseline characteristics

Baseline characteristics in our study (n = 322, 145 males and 177 females, median age (IQR) = 65 (55, 72) years) were demonstrated in Table 1. The median (IQR) serum Zn level for all cases was 73.2 (64.3, 81.7) μg/dl. LC was identified in 121 patients (37.6%). The median (IQR) serum Zn level in non-LC patients (75.8 (70.45, 84) μg/dl) was significantly higher than that in the LC group (64.3 (53.55, 74.6) μg/dl) (*P* < .0001). The median (IQR) BDI-II score and PSQI-J score were 6 (3, 12) and 5 (3, 7), respectively. There were 280 and 42 in groups of A (BDI-II score, 0–16 point) and B (BDI-II score, ≥17 point). There were 266 and 56 patients in groups of C (PSQI-J score, 0–8 point) and D (PSQI-J score, ≥9 point). In terms of SF-36, the median (IQR) PF, RP, BP, GH, VT, SF, RE, MH, PCS score and MCS score were 90 (80, 95), 100 (75, 100), 80 (52, 100), 53.3 (45, 67), 62.5 (50, 81.3), 100 (75, 100), 100 (75, 100), 80 (60, 90), 50.9 (42.25, 54.45) and 52.2 (44.05, 58.75), respectively.

**Table 1:**
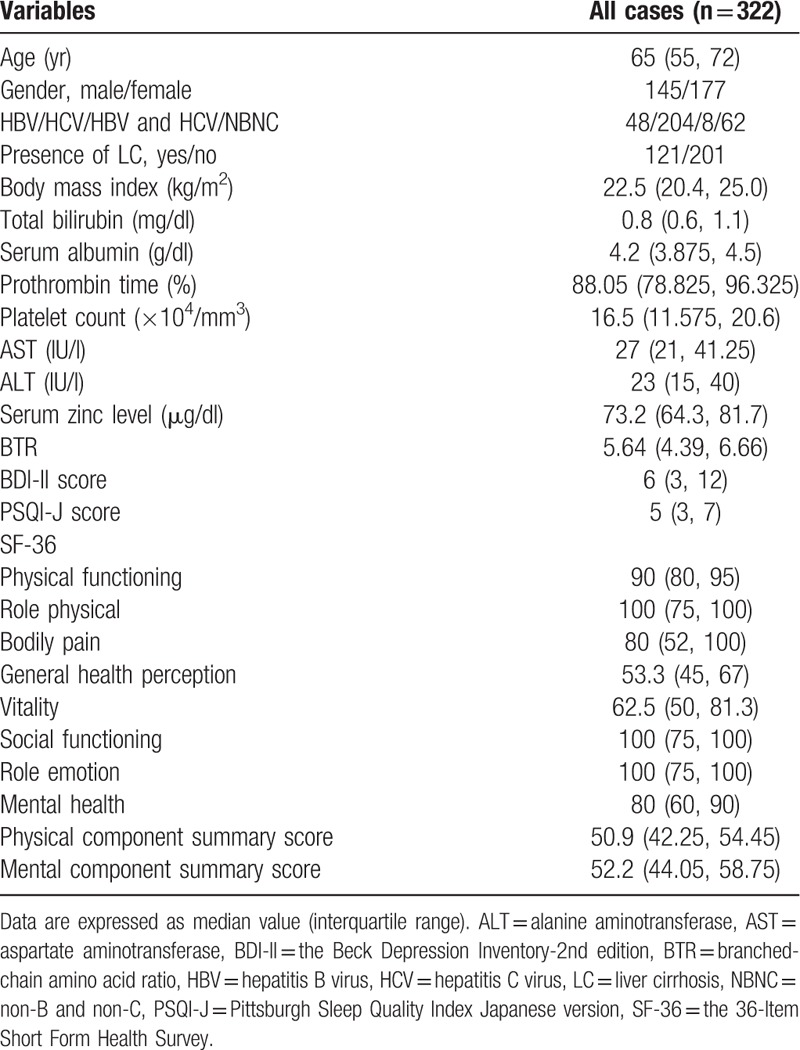
Baseline characteristics.

### 3.2 Correlation between serum Zn level and BDI-II score and PSQI-J score for all cases

**Figure 1:**
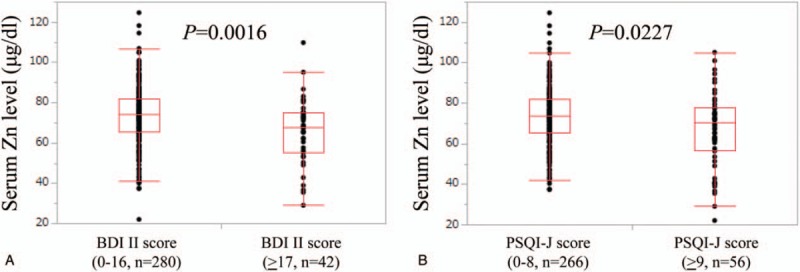
Correlation between serum Zn level and BDI-II score and PSQI-J score for all cases.

Patients with group A (n = 280: median (IQR) serum Zn level, 73.95 (65.5, 82.075) μg/dl) had significantly higher serum Zn level than those with group B (n = 42: median (IQR) serum Zn level, 67.45 (55.175, 74.925) μg/dl) (*P* = .0016), while patients with group C (n = 266: median (IQR) serum Zn level, 73.6 (65.5, 81.295) μg/dl) had significantly higher serum Zn level than those with group D (n = 56: median (IQR) serum Zn level, 70.45 (56.625, 77.875) μg/dl) (*P* = .0227). (Fig. 1A and B)

### 3.3 Correlation between serum Zn level and BDI-II score and PSQI-J score according to age

**Figure 2:**
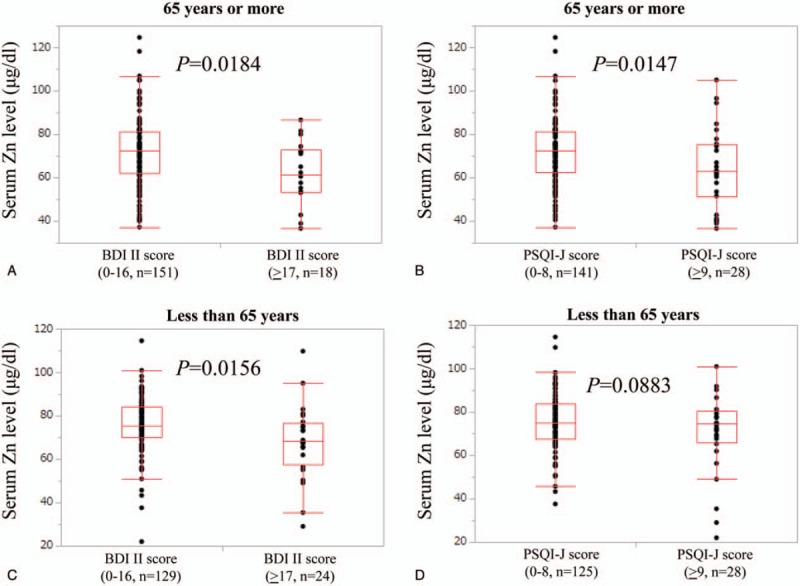
Correlation between serum Zn level and BDI-II score and PSQI-J score according to age.

In patients aged 65 years or older, patients with group A (n = 151: median (IQR) serum Zn level, 72.4 (62.2, 81.3) μg/dl) had significantly higher serum Zn level than those with group B (n = 18: median (IQR) serum Zn level, 61.4 (53.375, 72.825) μg/dl) (*P* = .0184), while patients with group C (n = 141: median (IQR) serum Zn level, 72.4 (62.45, 81.25) μg/dl) had significantly higher serum Zn level than those with group D (n = 28: median (IQR) serum Zn level, 62.85 (51.35, 75.25) μg/dl) (*P* = .0147). (Fig. 2A and B) In patients less than 65 years, patients with group A (n = 129: median (IQR) serum Zn level, 75.4 (69.9, 84.0) μg/dl) had significantly higher serum Zn level than those with group B (n = 24: median (IQR) serum Zn level, 68.4 (57.7, 76.65) μg/dl) (*P* = .0156), whereas the difference of serum Zn level in patients with group C (n = 125: median (IQR) serum Zn level, 75.1 (67.6, 83.7) μg/dl) and that in group D (n = 28: median (IQR) serum Zn level, 74.4 (65.9, 80.575) μg/dl) tended to be significant (*P* = .0883). (Fig. 2C and D)

### 3.4 Correlation between serum Zn level and BDI-II score and PSQI-J score according to liver disease etiology

**Figure 3:**
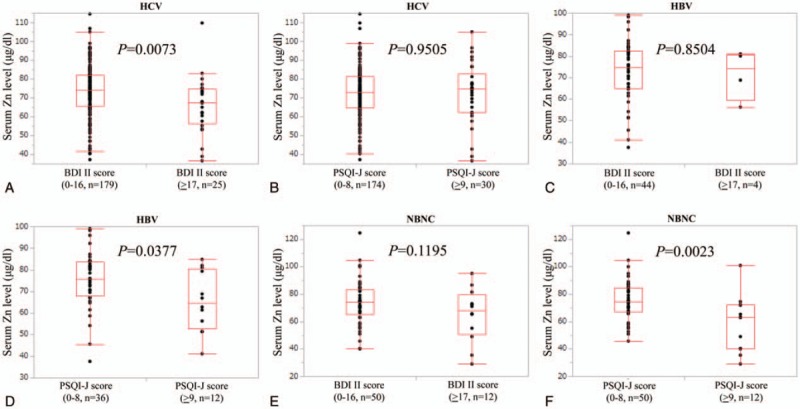
Correlation between serum Zn level and BDI-II score and PSQI-J score according to liver disease etiology.

In patients with hepatic C virus (HCV), patients with group A (n = 179: median (IQR) serum Zn level, 73.9 (65.5, 81.9) μg/dl) had significantly higher serum Zn level than those with group B (n = 25: median (IQR) serum Zn level, 67.2 (56.4, 74.6) μg/dl) (*P* = .0073), while patients with group C (n = 174: median (IQR) serum Zn level, 73.15 (64.65, 81.225) μg/dl) did not have significantly higher serum Zn level than those with group D (n = 30: median (IQR) serum Zn level, 74.75 (62.2, 82.5) μg/dl) (*P* = .9505). (Fig. 3A and B) In patients with hepatitis B virus (HBV), patients with group A (n = 44: median (IQR) serum Zn level, 74.7 (64.9, 82.6) μg/dl) did not have significantly higher serum Zn level than those with group B (n = 4: median (IQR) serum Zn level, 74.45 (59.425, 80.775) μg/dl) (*P* = .8504), whereas the difference of serum Zn level in patients with group C (n = 36: median (IQR) serum Zn level, 75.9 (67.775, 83.55) μg/dl) and that in group D (n = 12: median (IQR) serum Zn level, 64.85 (52.7, 80.575) μg/dl) reached significance (*P* = .0377). (Fig. 3C and D) In nonB and nonC (NBNC) patients, the difference of serum Zn level in patients with group A (n = 50: median (IQR) serum Zn level, 73.9 (65.525, 83.1) μg/dl) and that in group B (n = 12: median (IQR) serum Zn level, 68.35 (50.525, 79.375) μg/dl) did not reach significance (*P* = .1195), whereas patients with group C (n = 50: median (IQR) serum Zn level, 74.45 (67.05, 84.4) μg/dl) had significantly higher serum Zn level than those with group D (n = 12: median (IQR) serum Zn level, 63.05 (40.075, 72.2) μg/dl) (*P* = .0023). (Fig. 3E and F)

### 3.5 Correlation between serum Zn level and BDI-II score and PSQI-J score according to the LC status

**Figure 4:**
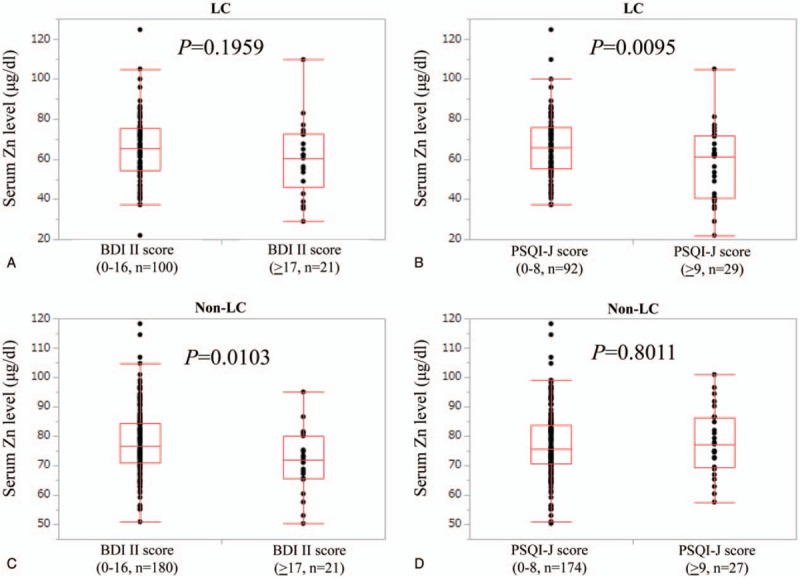
Correlation between serum Zn level and BDI-II score and PSQI-J score according to the LC status.

In LC patients, patients with group A (n = 100: median (IQR) serum Zn level, 65.5 (54.4, 75.375) μg/dl) did not have significantly higher serum Zn level than those with group B (n = 21: median (IQR) serum Zn level, 60.5 (45.9, 72.65) μg/dl) (*P* = .1959), whereas the difference of serum Zn level in patients with group C (n = 92: median (IQR) serum Zn level, 65.85 (55.325, 757.9) μg/dl) and that in group D (n = 29: median (IQR) serum Zn level, 61.4 (40.7, 71.65) μg/dl) reached significance (*P* = .0095). (Fig. 4A and B)

In non-LC patients, patients with group A (n = 180: median (IQR) serum Zn level, 76.5 (70.825, 84.475) μg/dl) had significantly higher serum Zn level than those with group B (n = 21: median (IQR) serum Zn level, 71.9 (65.5, 80.05) μg/dl) (*P* = .0103), whereas the difference of serum Zn level in patients with group C (n = 174: median (IQR) serum Zn level, 75.65 (70.475, 83.85) μg/dl) and that in group D (n = 27: median (IQR) serum Zn level, 77.3 (69.5, 86.4) μg/dl) did not reach significance (*P* = .8011). (Fig. 4C and D)

### 3.6 Correlation between serum zinc level and SF-36 for all cases

Correlation coefficients and *P* values between serum zinc level and data for SF-36 for all cases were listed in Table 2. PF, RP, BP, GH, VT, SF, RE, and PCS significantly correlated with serum Zn level.

**Table 2:**
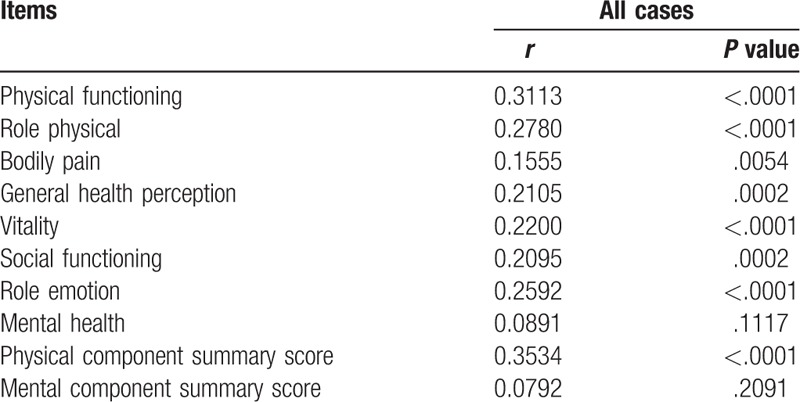
Correlation between serum zinc level and SF-36 for all cases (n = 322).

### 3.7 Correlation between serum zinc level and SF-36 according to age

Correlation coefficients and *P* values between serum zinc level and data for SF-36 according to age were listed in Table 3. In patients aged 65 years or more, PF, RP, BP, GH, VT, SF, RE, and PCS significantly correlated with serum Zn level, while in patients aged less than 65 years, PF, RP, GH, VT, SF, and PCS significantly correlated with serum Zn level.

**Table 3:**
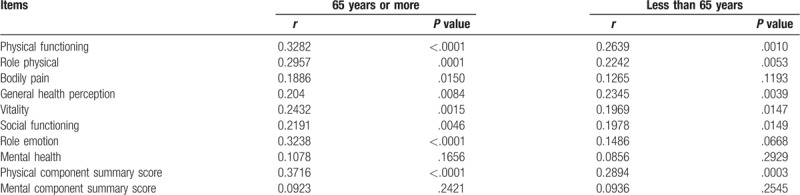
Correlation between serum zinc level and SF-36 according to age.

### 3.8 Correlation between serum zinc level and SF-36 according to liver disease etiology

Correlation coefficients and *P* values between serum zinc level and data for SF-36 according to liver disease etiology were listed in Table 4. In patients with HCV, PF, RP, GH, VT, SF, RE, and PCS significantly correlated with serum Zn level. In patients with HBV, PF, RP, GH, RE, and PCS significantly correlated with serum Zn level. In NBNC patients, PF, RP, BP, VT, SF, RE, and PCS significantly correlated with serum Zn level.

**Table 4:**
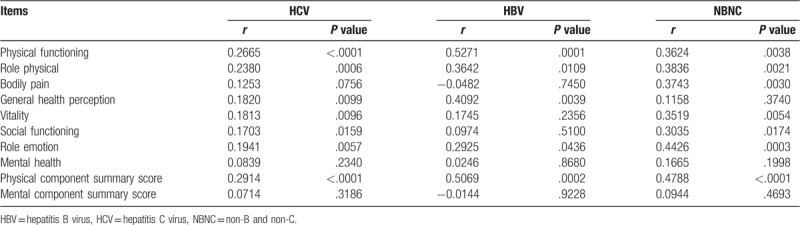
Correlation between serum zinc level and SF-36 according to liver disease etiology.

### 3.9 Correlation between serum zinc level and SF-36 according to the LC status

Correlation coefficients and *P* values between serum zinc level and data for SF-36 according to the LC status were listed in Table 5. In LC patients, PF, RP, BP, VT, RE, and PCS significantly correlated with serum Zn level, while in non-LC patients, PF, RP, and PCS significantly correlated with serum Zn level.

**Table 5:**
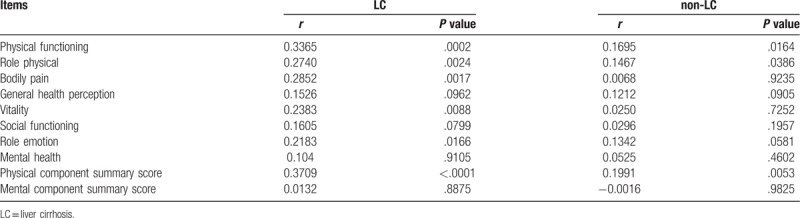
Correlation between serum zinc level and SF-36 according to the LC status.

## 4 Discussion

HRQOL has become established as an important concept and target for research and practice in the fields of medicine.^[32–34]^ Traditionally, not HRQOL but biochemical outcomes have been the primary endpoints in medical and health research, however, during the past decades, more researches have focused on patients’ HRQOL and the use of HRQOL evaluation has been increasing.^[34,35]^ In that sense, our current results appear to be worthy of reporting. A critique of HRQOL research is the lack of conceptual clarity and a common definition of HRQOL.^[36]^ We believe that using a clear definition of HRQOL may increase the conceptual understanding, which will help researches more rigorous. We thus used BDI-II score, PSQI-J score and SF-36 which are most widely used and validated assessment methods for the evaluation of QOL in the current analysis.^[19–22]^ These evaluation methods are also advantageous due to their excellence in quantitative properties for the evaluation of HRQOL.^[19–22]^

In our results, patients with higher BDI-II score tended to have lower serum Zn level compared with those with lower BDI-II score. Similar tendencies were observed in patients with higher PSQI-J score. As mentioned above, serum Zn loss can result in a wide spectrum of clinical manifestations and this can be linked to our current results.^[4,6–10]^ Not only hypozincemia but also worry about CLD disease progression can explain for depression or sleep disturbance.

More importantly, PF, RP, and PCS significantly correlated with serum Zn level regardless of age, liver disease etiology and LC status. While MH and MCS did not significantly correlate with serum Zn level regardless of age, liver disease etiology and LC status. These results gave us some insights for the better understanding of association of serum Zn level and HRQOL in CLDs. Lower PF indicates limited state in performing all physical activities and lower RP indicates problematic state with work or other daily activities as a result of physical health.^[22]^ Zn supplementation therapy may therefore improve these scores. While lower MH indicates feeling of nervousness and depression all of the time.^[22]^ Discrepancies exist between the association of serum Zn level and BDI-II score and MH or MCS in SF-36, although the reasons for these are unclear. Takuma, et al. reported in their RCT that in 79 LC patients with hepatic encephalopathy, Zn supplementation (225 mg of polaprezinc in addition to standard treatments for 6 months) significantly improved the PCS (*P* = .04), but not the MCS (*P* = .95).^[37]^ Their results may be associated with our data.

In patients with serum Zn level <60 μg/dl (n = 59), 10 patients (17.0%) were non-LC patients in our results. Japanese CLD patients are aging these days.^[38–41]^ There was a significant negative correlation between serum Zn level and age in our cohort (*r* = −0.1468, *P* = .0083). Even in non-LC patients, routine monitoring for serum Zn level may be mandatory.

Several limitations warrant mention to this study. First, the study was a single-center observational study with a retrospective nature. Second, serum Zn level can vary depending on patient daily life activities or dietary habits. Serum zinc level can be influenced by diurnal variation or fasting.^[42]^ Third, it was uncertain as to whether decreased HRQOL caused Zn loss or whether Zn loss caused decreased HRQOL in this cross-sectional study. Consequently, caution should be applied for the interpretation of our data. Despite these limitations, our study results denoted that Zn loss in CLDs was closely linked to decreased HRQOL. In conclusion, serum Zn level can be a useful marker for decreased HRQOL in patients with CLDs.

## Acknowledgment

The authors gratefully thank all medical staff in our nutritional guidance room for their help with data collection.

## Author contributions

**Data curation**: Hiroki Nishikawa, Kazunori Yoh, Yoshinori Iwata, Yoshiyuki Sakai, Kyohei Kishino, Naoto Ikeda, Tomoyuki Takashima, Nobuhiro Aizawa, Ryo Takata, Kunihiro Hasegawa, Noriko Ishii, Yukihisa Yuri, Takashi Nishimura and Hiroko Iijima. **Formal analysis**: Hiroki Nishikawa; **Supervision**: Shuhei Nishiguchi; **Writing – original draft**: Hiroki Nishikawa; **Writing – review & editing**: Hiroki Nishikawa, Hirayuki Enomoto and Shuhei Nishiguchi.
